# Parallel MapReduce: Maximizing Cloud Resource Utilization and Performance Improvement Using Parallel Execution Strategies

**DOI:** 10.1155/2018/7501042

**Published:** 2018-10-17

**Authors:** Ahmed Abdulhakim Al-Absi, Najeeb Abbas Al-Sammarraie, Wael Mohamed Shaher Yafooz, Dae-Ki Kang

**Affiliations:** ^1^Department of Smart Computing, Kyungdong University, Global Campus, 46 4-gil, Gosung, Gangwondo 24764, Republic of Korea; ^2^Faculty of Computer and Information Technology, Al-Madinah International University, 2 Jalan Tengku Ampuan Zabedah E/9E, 40100 Shah Alam, Selangor, Malaysia; ^3^Department of Computer & Information Engineering, Dongseo University, 47 Jurye-ro, Sasang-gu, Busan 47011, Republic of Korea

## Abstract

MapReduce is the preferred cloud computing framework used in large data analysis and application processing. MapReduce frameworks currently in place suffer performance degradation due to the adoption of sequential processing approaches with little modification and thus exhibit underutilization of cloud resources. To overcome this drawback and reduce costs, we introduce a Parallel MapReduce (*PMR*) framework in this paper. We design a novel parallel execution strategy of Map and Reduce worker nodes. Our strategy enables further performance improvement and efficient utilization of cloud resources execution of Map and Reduce functions to utilize multicore environments available with computing nodes. We explain in detail makespan modeling and working principle of the *PMR* framework in the paper. Performance of *PMR* is compared with Hadoop through experiments considering three biomedical applications. Experiments conducted for BLAST, CAP3, and DeepBind biomedical applications report makespan time reduction of 38.92%, 18.00%, and 34.62% considering the *PMR* framework against Hadoop framework. Experiments' results prove that the *PMR* cloud computing platform proposed is robust, cost-effective, and scalable, which sufficiently supports diverse applications on public and private cloud platforms. Consequently, overall presentation and results indicate that there is good matching between theoretical makespan modeling presented and experimental values investigated.

## 1. Introduction

Delivery model of data intensive applications/services on cloud platforms is the new paradigm. Scalable storage and computing capabilities of cloud platforms aid delivery models with various aspects. The cloud is maintained using distributed computing frameworks capable of handling and processing a large amount of data. Of all cloud frameworks available [1–5], Hadoop MapReduce is the most widely adopted [6, 7] owing to its ease of deployment, scalability, and open-source nature.

**Figure 1:**
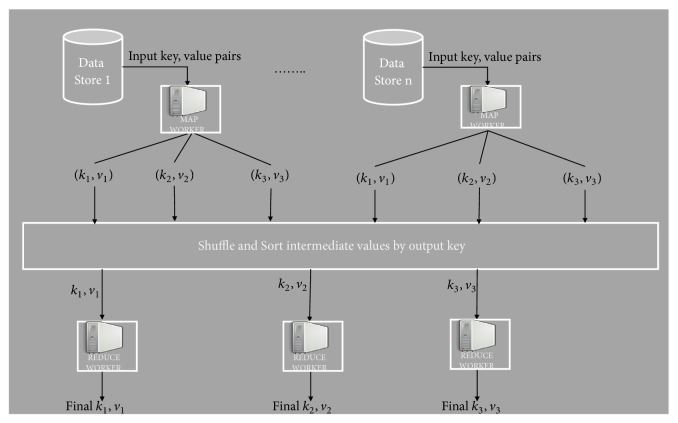
Hadoop MapReduce computation model.

The Hadoop MapReduce model predominantly consists of the following phases: Setup, Map, Shuffle, Sort, and Reduce, which is shown in Figure 1. The Hadoop frameworks consist of a master node and a cluster of computing nodes. Jobs submitted to Hadoop are further distributed into Map and Reduce tasks. In the Setup phase, input data of a job to be processed (residing generally on the Hadoop Distributed File Systems (HDFS)) is logically partitioned into homogenous volumes called chunks for the Map worker nodes. Hadoop divides each MapReduce job into a set of tasks where each chunk is processed by the Map worker. The Map phase takes input as key/value pair as (*k*_1_, *v*_1_) and generates a list of (*k*_2_, *v*_2_) intermediate key/value pairs as output. The Shuffle phase begins with completion of the Map phase that collects the intermediate key/value pairs from all the Map tasks. A Sort operation is performed on the intermediate key/value pairs of the Map phase. For simplicity, the Sort and Shuffle phases are cumulatively considered in the Shuffle phase. The Reduce phase processes sorted intermediate data based on user defined functions. The output of the Reduce phase is stored/written to HDFS.

The Hadoop MapReduce platform suffers from a number of drawbacks. The preconfigured memory allocator for Hadoop jobs leads to issues of buffer concurrency amongst jobs and heavy disk read seeks. The memory allocator issues result in increasing makespan time and induce high input/output (I/O) overheads [5]. The jobs scheduled on Hadoop cloud environments do not consider parameters such as memory requirement and multicore environment for linear scalability, which seriously affects performance [8]. In Hadoop, the Reduce tasks are started after completion of all Map tasks. Hadoop assumes homogenous Map execution times considering homogenous distributed data, which is not realistic [9]. Assumed homogenous Map execution times and serial execution strategy put forth utilized Map workers (and their resources) that have completed their tasks and are waiting for the other Map workers to complete theirs [10]. In cloud environments where organizations/users are charged according to (storage, computation, and communication) resources utilized, these issues burden the costs in addition to affecting performance [11]. Hadoop platforms do not support flexible pricing [12]. Scalability is an issue owing to the cluster based nature of Hadoop platforms. Processing of streaming data is also an issue with Hadoop [10]. To overcome these drawbacks, researchers have adopted various techniques.

In [5], they addressed the issues related to Hadoop memory management by adopting a global memory management technique. They proposed a prioritization model of memory allocation and revocation by adopting a rule based heuristic approach. A multithread execution engine is used to achieve global memory management. To address the garbage collection issue of a Java virtual machine (JVM) and to improve the data access rate in Hadoop, they adopted a multicache mechanism for sequential and interleaved disk access. Their model improves the memory utilization and balances the performance of I/O and CPU. In [5], the authors did not take the network I/O performance into consideration.

In [8], a GPU based model to address the linear scalability issue of Hadoop is presented. They addressed the research challenges of integrating Hadoop and GPU and how the MapReduce job can be executed using CUDA based GPU. In Hadoop MapReduce framework, the jobs run inside a JVM. Managing of jobs, creation of jobs, and executing of jobs suffer from computation overhead and reduce the efficiency of Just-In-Time (JIT) compilation due to the short-lived nature of jobs in the JVM. To overcome this, they adopted GPU based job execution approaches such as JNI, JCuda, Hadoop Pipes, and Hadoop Streaming. They have analyzed and evaluated detailed comparison of protocol of their pros and cons.

To address issues related to sequential execution in [13], a Cloud MapReduce (CMR) framework is discussed. Here, they developed a parallelized model by adopting a pipelining execution approach to process the streaming and batch data. Their cloud based MapReduce model supports parallelism between Map and Reduce phases and also among individual jobs.

The increased demand in data analytics for computing scientific/bioinformatics data has resulted in increased size of bioinformatics data. Computing and storing these huge data require a huge infrastructure. Computing bioinformatics application by adopting Cloud platform is a viable option for analyzing the genomic structure and its evolutionary pattern of large bioinformatics data [14–18] which is generated by the Next Generation Sequencing (NGS) technologies. Various cloud based bioinformatics applications have been developed to compute large bioinformatics data, CloudAligner [18], CloudBurst [19], Myrna [20], and Crossbow [21]. Cloud technologies allow the user to compute bioinformatics application and charges the user based on their usage. Reducing the computation cost in such environment is an area that needs to be considered during designing a bioinformatics computation model.

Reducing execution times and effective resource utilization with minimal costs are always a desired feature of cloud computing frameworks. To achieve this goal, a Parallel MapReduce (*PMR*) framework is proposed in this paper. The *PMR* adopts a parallel execution strategy similar to the technique presented in [13]. In conventional MapReduce systems, the Map phase is executed first, and then Reduce phase execution is considered. In the proposed PMR, Reduce phase execution is initiated in a parallel fashion, as soon as two or more Map worker nodes have completed their tasks. The adoption of such execution strategies enables reduction of unutilized worker resources. To further reduce makespan, parallel execution of the Map and Reduce functions is adopted utilizing multicore environments available with nodes. A makespan model to describe operations of the *PMR* is presented in future sections. Bioinformatics applications are synonymous with big data. Processing of such computationally heavy applications is considered on cloud platforms as investigated in [22, 23]. Performance evaluation of the PMR framework is carried out using bioinformatics applications. The major contributions can be summarized as follows:

- Makespan modeling and design of *PMR* cloud framework
- Parallel execution strategy of the Map and Reduce phase
- Maximizing cloud resource utilization by computing on multicore environments in Map and Reduce
- Performance evaluation on state-of-the-art biomedical applications like BLAST, CAP3, and DeepBind
- Experiments considering diverse cloud configurations and varied application configuration
- Correlation between theoretical makespan model and experimental values

Beside the data management and computing issues, there exist numerous security issues and challenges in provisioning security in cloud computing environment and in ensuring ethical treatment of biomedical data. When MapReduce is carried out in distributed settings, users maintain very little control over these computations, causing several security and privacy concerns. MapReduce activities may be subverted or compromised by malicious or cheating nodes. Such security issues have been discussed and highlighted by many researchers as in [24–26]. However, addressing security issues is beyond the scope of this paper.

The paper organization is as follows: In Section 2, the related works are discussed. In Section 3, the proposed *PMR* framework is presented. The results and the experimental study are presented in the penultimate section. The concluding remarks are discussed in the last section.

## 2. Literature Review

D. Dahiphale et al. [13] presented a cloud based MapReduce model to overcome the shortcomings of the Hadoop MapReduce model which are as follows: Hadoop processes the Map and Reduce phases in a sequential manner, scalability is not efficient due to cluster based computing mechanism, processing of stream data is not supported, and lastly it does not support flexible pricing. To overcome the issue of sequential execution, they proposed a cloud based Parallel MapReduce model where the tasks are executed by the Amazon EC2 instances (virtual machine (worker)); to process stream and batch data in a parallel manner, a pipelining model is adopted which provides flexible pricing by using an Amazon cloud Spot Instance. Experiment result shows that the CMR model processes tasks in a parallel manner, improves the throughput, and shows a speedup improvement of 30% over the Hadoop MapReduce model for larger datasets.

X. Shi et al. [5] presented a framework for memory intensive computing to overcome the shortcomings of the Hadoop MapReduce model. In Hadoop, tasks are executed based on the available CPU cores and memory is allocated based on a preset configuration which lead to memory bottleneck due to buffer concurrency and heavy disk seeks resulting in I/O wait occupancy which further increases the makespan time. To address this, they presented a rule based heuristic model to prioritize memory allocation and revocation for global memory management. They presented a multithread approach for which they developed disk access serialization, multicache technique for efficient garbage collection in JVM. The experimental study shows that execution of memory intensive computation time is improved by 40% over the Hadoop MapReduce model.

Babak Alipanahi et al. [27] presented a model by adopting deep learning techniques for DNA- and RNA-binding protein for pattern discovery. The specificity of protein is generally described using position weight matrices (PWMs) and the learning sequence specificity in the high throughput model has the following challenges. Firstly, there is the varied nature data from different sources. For example, chromatin immunoprecipitation provides varying putatively bound sequence length of ranked list, for each sequence, RNAcompete assay and protein binding microarray provide a specificity coefficient, and HT-SELEX produces a very high similarity sequence set. Secondly, each data provider has its unique biases, artifacts, and limitation for which it needs to identify the pertinent specificity. Lastly, the data are in huge size which requires a computation model to integrate all data from different sources. To overcome these challenges, they presented a model, namely, DeepBind, whose characteristics are as follows. It is applicable for both sequence and microarray data and works well across different technologies without correcting for technology-specific biases. Sequences are processed in a parallel manner by using a graphics processing unit (GPU), which can train the predicting model automatically and can withstand a modest degree of noisy and incorrectly labeled trained data. Experiments are conducted on in vitro data for both training and testing which shows that the DeepBind model is a scalable modular pattern discovery technique based on deep learning which does not depend on application specific heuristics such as “seed finding.”

K. Mahadik et al. [28] presented a parallelized BLAST model to overcome issues related to mpiBLAST which are as follows. It segments the database and processes each short query in parallel but due to rapid growth of NGS it has resulted in increased size of sequences (long query sequences) which can be millions of protein/nucleotide sequences which limits the mpiBLAST resulting in scheduling overhead and increasing the makespan time. The mpiBLAST task completion time of short queries is faster as compared to large queries which create improper load balancing among nodes. To address this, they presented a parallel model of BLAST, namely, ORION, splitting individual queries into overlapping fragments to process large query sequences on the Hadoop MapReduce platform. Experimental outcomes show that their model achieves a speedup of 12.3x over mpiBLAST without compromising on accuracy.

J. Ekanayake et al. [29] presented a cloud based MapReduce model, namely, Microsoft DryadLINQ and Apache Hadoop, for bioinformatics applications and it was compared with the existing MPI framework. The pairwise Alu sequence alignment and CAP3 [30] application is considered. To evaluate the scheduling performance of these frameworks, an inhomogeneous dataset is considered. Their outcomes show that two cloud frameworks have a significant advantage over MPI in terms of fault tolerance, parallel execution by adopting the MapReduce framework, robustness, and flexibility since MPI is memory based whereas the DryadLINQ and Hadoop model is file oriented based. Experimental analysis is conducted for varied sequence sizes and the result shows that Hadoop performs better than DryadLINQ for inhomogeneous data for both applications.

Y. Wu et al. [31] presented an outliers based execution strategy *NO*^2^ for computation intensive applications in order to reduce the makespan and overhead of computation; many existing approaches that adopt a MapReduce framework are suitable for data intensive application since their scheduler state is defined by I/O status. They designed a framework for computation intensive tasks by adopting instrumentation to detect task progress and automatic instrument point selector to reduce overhead and finally for outlier's detection without resorting to biased progress calculation K-means is adopted. The *NO*^2^ framework is evaluated by using application CAP3 and ImageMagick on both local cluster and cloud environment. Their threshold based outlier model improves the task completion time by 25% with minimal overhead.

## 3. The Proposed* PMR* Framework

The *PMR* framework incorporates similar functions available in conventional MapReduce frameworks. Accordingly, the Map, Shuffle (including Sort), and Reduce phases exist in *PMR*. For the sake of representation simplicity, the Shuffle and Reduce phases are cumulatively considered in the Reduce phase. The Map phase takes input data for processing and generates a list of key pair values of result *M*(*key*_1_, *val*_1_) → *l*(*key*_2_, *val*_2_). This generated key *key*_2_ and list of different values are integrated together and put into a reducer function. The reducer function takes intermediate key *key*_2_ and processes the values and generates a new set of values *l*(*val*_3_).

The *PMR* job execution is performed on multiple virtual machines forming a computing cluster, where one is a master node and the others are worker nodes/slave nodes. The master node distributes and monitors tasks among worker nodes. Worker nodes periodically send their resource utilization details to the master node. Master nodes schedule the task based on availability of worker resources.

To minimize makespan of job execution and maximize utilization of cloud resource (available with worker nodes), the proposed *PMR* adopts a parallel execution strategy; i.e., Reduce phase execution is initiated in a parallel fashion, as soon as two or more Map worker nodes have completed their tasks. The worker nodes are considered to have more than one computing core; the *PMR* framework presents parallel execution of the Map and Reduce functions adopted utilizing multicore environments and a makespan model of the proposed *PMR* is described and presented in Section 3.1.

The *PMR* function is a combination of the Map task and Reduce task. The input dataset is split into uniform block sized data called chunks and is distributed among the computing nodes. In *PMR*, the chunk obtained is further split to parallelize execution of user defined Map and Reduce functions. The user defined Map function is applied on the input and intermediate output is generated which is input data for the Reduce task. The Reduce stage is a combination of two phases, Shuffle and Reduce. Output data which is generated from the Map task is fed as an input in the Shuffle phase; the already completed Map task is shuffled and then sorted in this phase. Now, the sorted data is fed into the user defined Reduce function and the generated output is written back to cloud storage.

A Map function in terms of computation time and input/output data dependencies can be represented as a tuple

(1) $$\begin{array}{c} \left(\;{\vec{Size}}_{M}^{in},S_{M},M_{↓},M_{\to },M_{↑}\;\right), \end{array}$$

where ${\vec{Size}}_{ℳ}^{in}$ is the average input data processed by each Map worker. Variables *ℳ*_↓_, *ℳ*_→_  and  *ℳ*_↑_ represent the maximum, average, and minimum computation time of the Map function. Output of the Map function stored in the cloud to be processed by Reduce workers is represented as a ratio between output and input data *S*_*ℳ*_.

Similarly, the *PMR* Reduce function is represented as

(2) $$\begin{array}{c} \left(\;S_{M},S_{R},R_{↓},R_{\to },R_{↑}\;\right), \end{array}$$

where *S*_*ℳ*_ is output data of Map functions stored in the cloud (represented as a ratio). Output of the Reduce function or the task assigned to *PMR* is represented as *S*_*R*_ (ratio of Reduce output to input). Minimum, average, and maximum computation times of the Reduce function are *R*_↓_, *R*_→_  and  *R*_↑_. The Reduce stage incorporates Shuffle and Sort operations.

Reducing execution time and minimizing cost of cloud usage are always desirable attributes. In this paper, a makespan model to describe operation of the *PMR* framework is presented. Obtaining actual makespan times is very complex and is always a challenge. A number of dependencies exist, like hardware parameters, network conditions, cluster node performance, cloud storage parameters, data transfer rates, etc. in obtaining makespans. The makespan model of *PMR* described below only considers functional changes incorporated to improve performance in conventional MapReduce frameworks. Modeling described below is based on work presented in [32].

### 3.1. PMR Makespan Model

#### 3.1.1. Preliminaries and Makespan Bound Establishment

The makespan function is computed as the time required to complete a job of input data size and number of resources which is allocated to *PMR*. Let us consider a job *J* to be executed on the *PMR* cloud platform considering data *D*. Let the cloud platform have *n* + 1 number of nodes/workers. Each worker is said to have *p* cores that can be utilized for computation. One worker node acts as the master node leaving *n* number of workers to perform Map and Reduce computation. Job *J* is considered to be distributed and computed using an *x* number of Map and Reduce tasks. The data *D* is also accordingly split into *x* chunks represented as *D*′. In conventional MapReduce platforms, *D*′ = (*D*/*n*). PMR considers a similar approach for computing *D*′. The time utilized to complete *x* tasks is represented as *𝒯*_1_, *𝒯*_2_,…, *𝒯*_*x*_. In the proposed PMR framework, the chunks *D*′ are further split into *D*′′ = (*D*′/*p*) for parallel execution. In PMR execution of the *x*^*th*^ task, *𝒯*_*x*_ = *𝔗*_↑_ = max⁡{*𝔱*_1_, ⋯*𝔱*_*p*_}, where *𝔱*_*p*_ represents execution of task on the *p*^*th*^ core considering corresponding data *D*′′.

Average (*σ*) and maximum time (*β*) duration taken by *x* tasks to complete job *J* can be represented as

(3) σ=∑j=1xTjxβ=maxj⁡Tj.

Let us consider an optimistic scenario that *x* tasks are uniformly distributed among *q* worker nodes (minimum time taken to process (*x* × *σ*) work). Overall, the time taken to compute these tasks is (*x* × *σ*)/*q* and it is the lower bound time.

(4) $$\begin{array}{c} lb=\frac{x\times \sigma }{q}. \end{array}$$

To compute the upper bound time, a pessimistic scenario is considered, where the longest processing task $\overset{\leftarrow }{𝒯}\in ({𝒯}_{1},{𝒯}_{2},\ldots ,{𝒯}_{x})$ with makespan of *β* is the last processed task. Therefore, the time taken before the last task $\overset{\leftarrow }{𝒯}$ is upper bounded as follows:

(5) $$\begin{array}{c} \frac{\left(\sum_{j=1}^{x}T_{j}\right)}{q}\leq \frac{\left(x-1\right)\times \sigma }{q}. \end{array}$$

Therefore, the overall timespan for this longest task $\overset{\leftarrow }{𝒯}$ is upper bounded as ((*x* − 1) × *σ*)/*q* + *β*. The probable job makespan range due to nondeterminism and scheduling is obtained by the difference lower bound and upper bound. This is a key factor when the time taken of the longest task is trivial as compared to the overall makespan; i.e., *β* ≪ (*x* × *σ*/*q*).

(6) $$\begin{array}{c} ub=\frac{\left(x-1\right)\times \sigma }{q}+\beta . \end{array}$$

#### 3.1.2. Makespan of a Job on the PMR Framework

Let us consider a job *𝒥* which is submitted to the *PMR* framework; the job *𝒥* is split into *X*_*ℳ*_^*𝒥*^ number of Map tasks and *X*_*R*_^*𝒥*^ number of Reduce tasks. Let *𝒮*_*ℳ*_^*𝒥*^ and *𝒮*_*ℛ*_^*𝒥*^ represent the number of Map and Reduce workers allocated for the *𝒥*^*th*^ job.

To compute the makespan of the Map tasks, the lower and upper bounds are computed. Using (3), average (*ℳ*_→_) and maximum (*ℳ*_↑_) makespan of Map tasks of job *𝒥* are computed. Using *ℳ*_↑_,and *ℳ*_→_ computed in (4), the lower bound of the Map phase, i.e., *𝒯*_*M*_^*lb*^, is defined as

(7) $$\begin{array}{c} T_{M}^{lb}=\frac{X_{M}^{J}\times M_{\to }}{S_{M}^{J}}. \end{array}$$

Similarly, the upper bound *𝒯*_*M*_^*ub*^ or the maximum execution time of the Map phase in *PMR* using (6) is defined as

(8) $$\begin{array}{c} T_{M}^{ub}=\frac{\left(X_{M}^{J}-1\right)\times M_{\to }}{S_{M}^{J}+M_{↑}}. \end{array}$$

Considering the lower (*𝒯*_*M*_^*lb*^) and upper (*𝒯*_*M*_^*ub*^) bounds computed, the makespan of the Map phase in *PMR* is computed as

(9) $$\begin{array}{c} {\vec{T}}_{M}=\frac{\left(T_{M}^{ub}+T_{M}^{lb}\right)}{2}. \end{array}$$

The average makespan of each Map worker node is computed as

(10) $$\begin{array}{c} {\vec{T}}_{M⟶}=\frac{{\vec{T}}_{M}}{S_{M}^{J}}. \end{array}$$

**Figure 2:**
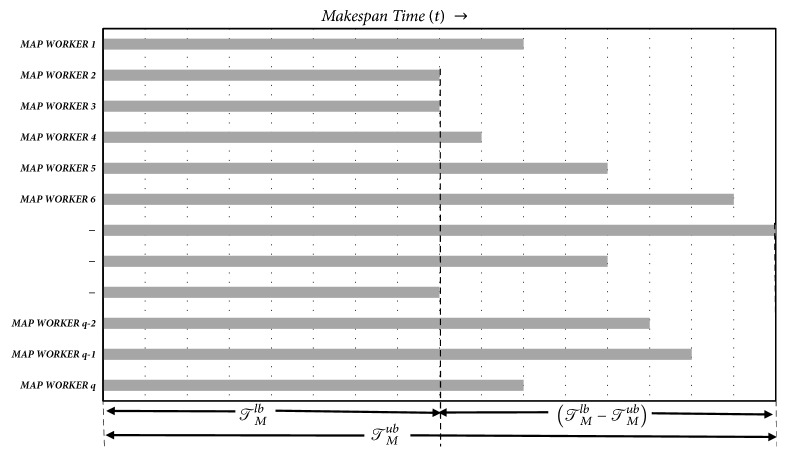
Map phase makespan of PMR framework.

The makespan of the PMR Map phase consisting of *𝒮*_*ℳ*_^*𝒥*^ = *q* worker nodes is shown in Figure 2 of the paper. Ascertaining bounds of makespan, i.e., *𝒯*_*M*_^*ub*^ and *𝒯*_*M*_^*lb*^, is shown in the figure.

The Reduce workers are initiated when at least two Map worker nodes have finished their computational tasks. The Reduce phase is initiated at (*𝒯*_*M*_^*ub*^ − *𝒯*_*M*_^*lb*^) time instance. Intermediate data generated by Map worker nodes is processed using the Shuffle, Sort, and Reduce functions defined. Average execution time *ℛ*_→_ and maximum execution time *ℛ*_↑_ of the Reduce phase considering *𝒮*_*ℛ*_^*𝒥*^ workers are derived using (3). Makespan bounding of the Reduce phase is computed (the lower bound is represented as *𝒯*_*R*_^*lb*^ and the upper bound is represented as *𝒯*_*R*_^*ub*^) as follows:

(11) TRlb=XRJ∙R→SRJ

(12) TRub=XRJ−1∙R→SRJ+R↑.

The makespan of the *𝒥*^*th*^ job on the *PMR* framework is a sum of time taken to execute Map tasks and time taken to execute Reduce tasks. Considering the best case scenario (lower bound), the minimum makespan observed is

(13) $$\begin{array}{c} T_{J}^{lb}=T_{M}^{lb}+T_{R}^{lb}-\left(\;T_{M}^{ub}-T_{M}^{lb}\;\right). \end{array}$$

Simplifying (13), we get

(14) $$\begin{array}{c} T_{J}^{lb}=2T_{M}^{lb}+T_{R}^{lb}-T_{M}^{ub}. \end{array}$$

Considering the worst computing performance, the upper bound or maximum makespan observed is

(15) TJub=TMub+TRub−TMub−TMlb

(16) TJub=TMlb+TRub.

The makespan of job *𝒥* on the *PMR* framework is defined as

(17) $$\begin{array}{c} {\vec{T}}_{J}=\frac{\left(T_{J}^{ub}+T_{J}^{lb}\right)}{2}. \end{array}$$

Using (14) and (16), makespan *𝒥* is

(18) T→J=TMlb+TRub+2TMlb+TRlb−TMub2=3TMlb+TRlb+TRub−TMub2.

#### 3.1.3. Modeling Data Dependency on Makespan

According to [30, 31], data dependency can be modeled using linear regression. A similar approach is adopted here. The average makespan of the *x*^*th*^ Map worker node is defined as

(19) $$\begin{array}{c} M_{\to }^{x}=V_{0}^{M}+\overset{S_{M}^{J}}{\underset{w=1}{\sum }}\left(\;V_{w}^{M}\left(\;\frac{D^{\prime }}{p}\;\right)\;\right), \end{array}$$

where *𝒱*_*∗*_^*M*^ represent variables that are application specific; i.e., they are dependent on the Map user function.

The average makespan of the *x*^*th*^ Reduce worker node is defined as

(20) $$\begin{array}{c} R_{\to }^{x}=V_{0}^{R}+\overset{S_{R}^{J}}{\underset{w=1}{\sum }}\left(\;V_{w}^{R}\left(\;\frac{d^{\prime }}{p}\;\right)\;\right), \end{array}$$

where *𝒱*_*∗*_^*R*^ represent variables specific to the user defined Reduce functions and *d*′represents intermediate output data obtained from the Map phase. For parallel execution and to utilize all resources, it is further split similar to the Map phase.

On similar lines, the average and maximum execution times of Map and Reduce workers are computed. Data dependent computations of *ℳ*_→_, *ℳ*_↑_, *ℛ*_→_, *ℛ*_↑_ are used in (14), (16), and (18) to compute makespan of the *𝒥*^*th*^ job on the *PMR* framework considering data *D*. Additional details of data dependency modeling using linear regression are presented in [33]. The proof of the model is also presented in [33].

## 4. Performance Evaluation

Experiments conducted to evaluate the performance of *PMR* are presented in this section. Performance of *PMR* is compared with the state-of-the-art Hadoop framework. Hadoop is the most widely used/adopted MapReduce platform for computing in cloud environments [34]; hence, it is considered for comparisons. The *PMR* framework is developed using VC++, C#, and Node.js and deployed on the Azure cloud. Hadoop 2, i.e., version 2.6, is used and deployed on the Azure cloud using HDInsight. The *PMR* framework is deployed consisting of one master node and 4 worker nodes. Each worker node is deployed on A3 virtual machine instances. Each A3 VM instance consists of 4 virtual computing cores, 7 GB of RAM, and 120 GB of local hard drive space. The Hadoop platform deployed for evaluation consists of one master and 4 worker nodes in the cluster. Uniform configuration of *PMR* and Hadoop frameworks on Azure cloud is considered.

Biomedical applications characterized by processing of massive amounts of genetic data are considered in the experiments for performance evaluation. A computationally heavy biomedical application, namely, BLAST [35], CAP3 [30], and state-of-the-art recent DeepBind [27], is adopted for evaluation. All the genomic sequences considered for the experimental analyses are obtained from the publicly available NCBI database [36]. For comprehensive performance evaluations, the authors have considered various application scenarios. In experiments conducted using BLAST, both the Map and Reduce phases are involved. In CAP3 application, the Map phase plays a predominant role. In DeepBind, the Reduce phase is critical for analysis.

### 4.1. BLAST

Gene sequence alignment is a fundamental operation adopted to identify similarities that exist between a query protein sequence, DNA or RNA, and a database of sequences maintained. Sequence alignment is computationally heavy and its computation complexity is relative to the product of two sequences being currently analyzed. Massive volumes of sequences maintained in the database to be searched induce an additional computation burden. BLAST is a widely adopted bioinformatics tool for sequence alignment which performs faster alignments, at the expense of accuracy (possibly missing some potential hits) [35]. The drawbacks of BLAST and its improvements are discussed in [28]. For evaluation here, the improved BLAST algorithm of [28] is adopted. To improve computation time, a heuristic strategy is used compromising accuracy minimally. In the heuristic strategy, an initial match is found and is later extended to obtain the complete matching sequence.

A three-stage approach is adopted in BLAST for sequence alignment. Query sequence is represented using *𝓆* and reference sequence as *𝓇*. Sequences *𝓆* and *𝓇* are said to consist of *k*−length subsequences known as *k* − *mers*. In the initial stage, also known as the *k* − *mer* match stage, BLAST considers each of the *k* − *mers* of *𝓆* and *𝓇* and searches for *k* − *mers* that match in both. This process is repeated to build a scanner of all *k*−letter words in query *𝓆*. Then, BLAST searches reference genome *𝓇* by using the scanner built to find *k* − *mers* of *𝓇* matches with query *𝓆* and these matches are *seeds* of potential hits.

In the second stage, also known as the ungapped alignment stage, every seed identified previously is *extended* in both directions, respectively, to include matches and mismatches. A match is found if nucleotides in *𝓆* and *𝓇* are the same. A mismatch occurs if varied nucleotides are observed in *𝓆* and *𝓇*. The mismatch reduces the score and matches increase the score of candidate sequence alignment. The present score of sequence alignment *a* and the highest score obtained for present seed *a*^↑^ are retained. The second phase is terminated if *a*^↑^ − *a* is higher than the predefined X-drop threshold *h*_*x*_ and returns with the highest alignment score of the present seed. The alignment is passed to stage three if the returned score is higher than the predefined ungapped threshold *H*_*y*_. The thresholds predefined establish accuracy of alignment scores in BLAST. Computational optimization is achieved by skipping seeds already available in previous alignments. The initial two phases of BLAST are executed in the Map workers of *PMR* and Hadoop.

**Figure 3:**
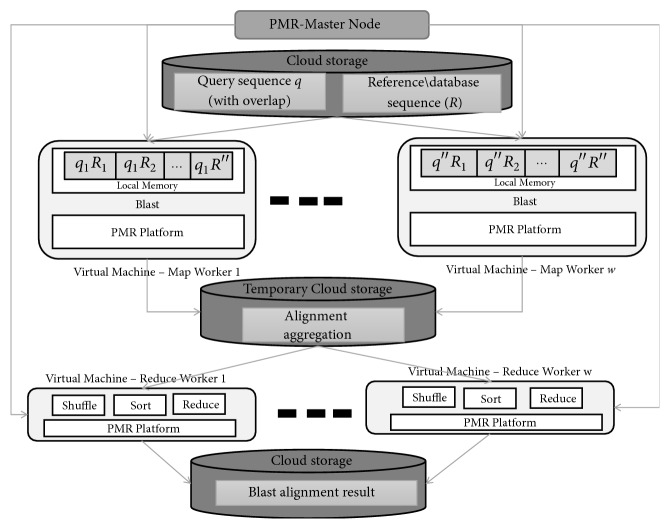
BLAST PMR framework.

In stage three, gapped alignment is performed in the left and right directions where deletion and insertion are performed during extension of alignments. The same as the previous stage, the highest score of alignment *a*^↑^ is kept and if the present score *a* is lower than *a*^↑^ by more than the X-drop threshold, the stage is terminated and the corresponding alignment outcome is obtained. Gap alignment operation is carried out in the Reduce phase of *PMR* and Hadoop framework. The schematic of BLAST algorithm on *PMR* framework is shown in Figure 3.

Experiments conducted to evaluate performance of *PMR* and Hadoop considered the* Drosophila* database as a reference database. The query genomics of varied sizes considered is from* Homo sapiens* chromosomal sequences and genomic scaffolds. A total of six different query sequences are considered similar to [28]. Configuration of each experiment is summarized in Table 1. All six experiments are conducted using BLAST algorithm on Hadoop and *PMR* frameworks. All observations retrieved through a set of log files generated during the Map and Reduce phases of Hadoop and *PMR* are noted and stored for further analysis. Using the log files total makespan, Map worker makespan, and Reduce worker makespan of Hadoop and *PMR* is noted for each experiment. It must be noted that the initialization time of the VM cluster is not considered in the computing makespan as it is uniform in *PMR* and Hadoop owing to similar cluster configurations.

**Figure 4:**
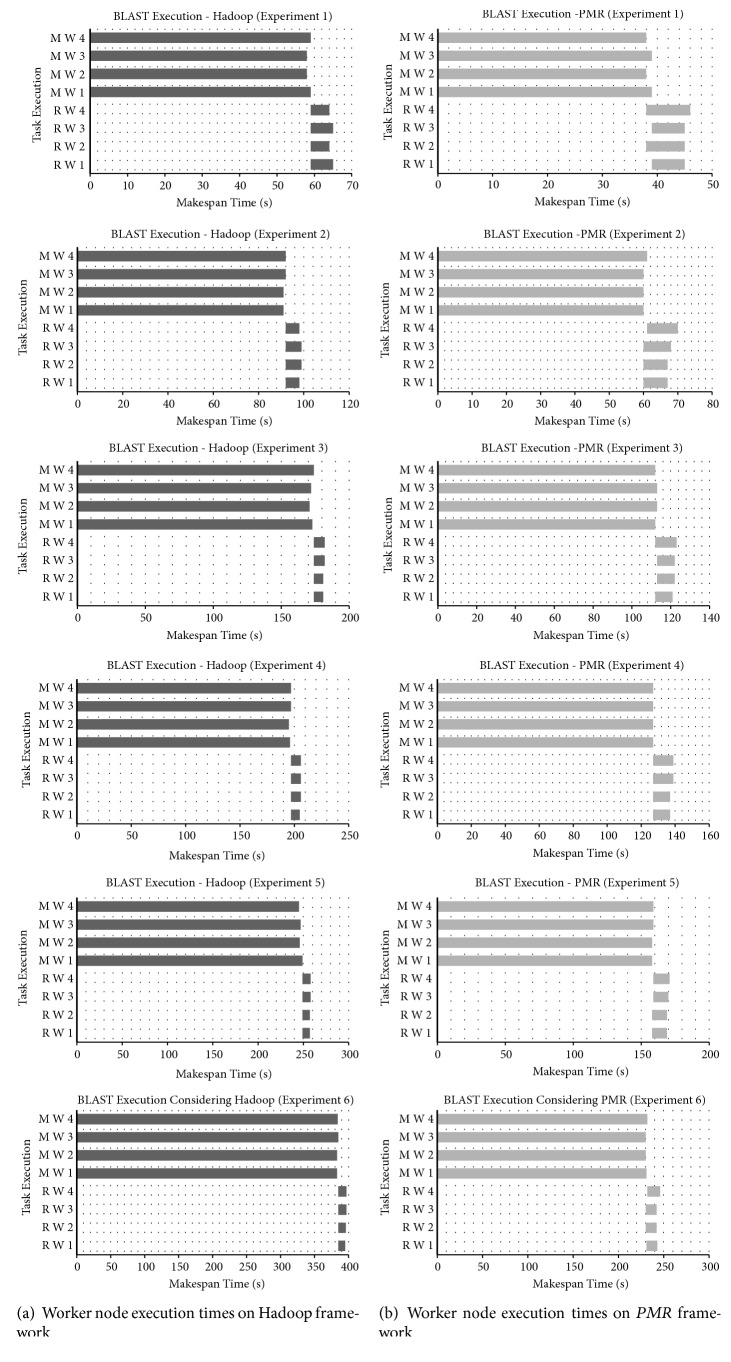
BLAST sequence alignment execution makespan of the Map and Reduce worker nodes. (a) On Hadoop cluster of 4 nodes. (b) On *PMR* cluster of 4 nodes.

Individual task execution times of Map worker and Reduce worker nodes observed for each BLAST experiment executed on Hadoop and *PMR* frameworks are graphically shown in Figure 4.  Figure 4(a) represents results obtained for Hadoop and Figure 4(b) represents results obtained on *PMR*. Execution times of Map workers in *PMR* and Hadoop are dominantly higher than Reduce worker times. This is due to the fact that major computation intensive phases (i.e., Phase 1 and Phase 2) of BLAST sequence alignment application are carried out in the Map phase. Parallel execution of BLAST sequence alignment utilizing all 4 cores available with each Map worker node adopted in *PMR* results in lower execution times when compared to Hadoop Map worker nodes. Average reduction of execution time in Map workers of *PMR* is 34.19%, 34.15%, 34.78%, 35.29%, 35.76%, and 39.87% in experiments conducted when compared to Hadoop Map worker average execution times. As the query size increases, performance improvement of *PMR* increases. Parallel execution strategy of Reduce worker nodes proposed in *PMR* is clearly visible in Figure 4(b). In other words, Reduce workers are initiated as soon as two or more Map worker nodes have completed their tasks. The execution time of Reduce worker nodes in *PMR* is marginally higher than those of Hadoop. Waiting for all Map worker nodes to complete their tasks is a primary reason for the marginal increase in Reduce worker execution times in *MR*. Sequential processing, i.e., Map workers first and then Reduce worker execution, of worker nodes in Hadoop framework is evident from Figure 4(a).

**Figure 5:**
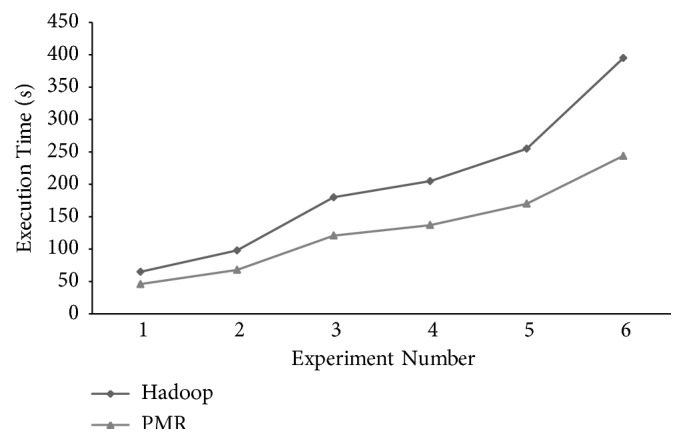
BLAST sequence alignment total makespan time observed for experiments conducted on *PMR* and Hadoop frameworks.

The total makespan of *PMR* and Hadoop is dependent on task execution time of worker nodes during the Map phase and Reduce phase. The total makespan observed in BLAST sequence alignment experiments executed on Hadoop and *PMR* frameworks is shown in Figure 5. Superior performance in terms of Reduce makespan times of *PMR* is evident when compared to Hadoop. Though a marginal increase in Reduce worker execution time is reported, overall execution time, i.e., total makespan of *PMR*, is less when compared to Hadoop. A reduction of 29.23%, 30.61%, 32.78%, 33.17%, 33.33%, and 38.23% is reported for six experiments executed on the *PMR* framework when compared to similar experiments executed on Hadoop framework. Average reduction of the total makespan across all experiments is 32.89% proving superior performance of *PMR* when compared to Hadoop framework.

**Figure 6:**
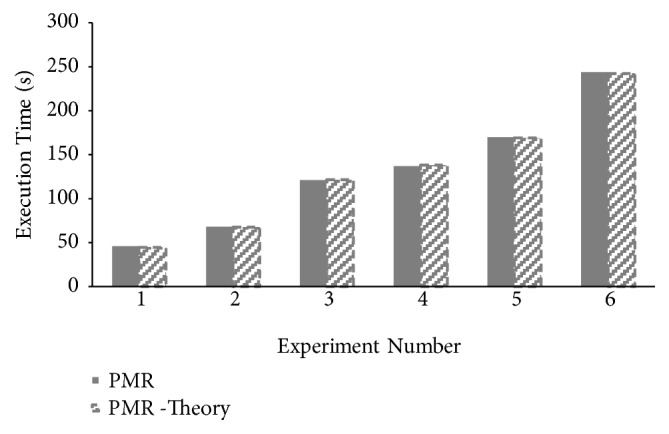
Correlation between theoretical and practical makespan times for BLAST sequence alignment execution on PMR framework.

Theoretical makespan of *PMR*, *i*.*e*., *𝒥*, given by (18) is computed and compared against the practical values observed in all the experiments. Results obtained are shown in Figure 6. Minor variations are observed between practical and theoretical makespan computations. Overall good correlation is reported between practical makespan values and theoretical makespan values. Based on the results presented, it is evident that execution of BLAST sequence alignment algorithm on the proposed *PMR* yields superior results when compared to similar experiments conducted on the existing Hadoop framework. Accuracy and correctness of the theoretical makespan model of *PMR* presented are proved through correlation measures.

### 4.2. CAP3

DNA sequence assembly tools are used in bioinformatics for gene discovery and understanding genomes of existing/new organisms. CAP3 is one such popular tool used to assemble DNA sequences. DNA assembly is achieved by performing merging and aligning operations on smaller sequence fragments to build complete genome sequences. CAP3 eliminates poor sections observed within DNA fragments, computes overlaps amongst DNA fragments, is capable of identifying false overlaps, eliminating false overlaps identified, accumulates fragments of multiple or one overlapping DNA segment to produce contigs, and performs multiple sequence alignments to produce consensus sequences. CAP3 reads multiple gene sequences from an input FASTA file and generates output consensus sequences written to multiple files and also to standard outputs.

**Figure 7:**
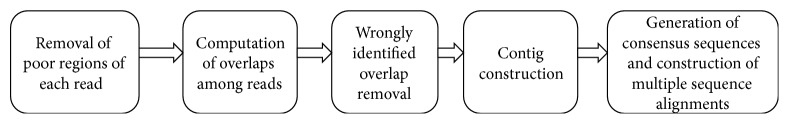
Steps for CAP3 sequencing assembly.

The CAP3 gene sequence assembly working principle consists of the following key stages. Firstly, the poor regions of 3′ (three-prime) and 5′ (five-prime) of each read are identified and eliminated. False overlaps are identified and eliminated. Secondly, to form contigs, reads are combined based on overlapping scores in descending order. Further, to incorporate modifications to the contigs constructed, forward-reverse constraints are adopted. Lastly, numerous sequence alignments of reads are constructed per contig resulting in consensus sequences characterized by a quality value for each base. Quality values of consensus sequences are used in construction of numerous sequence alignment operations and also in computation of overlaps. Operational steps of CAP3 assembly model are shown in Figure 7. A detailed explanation of the CAP3 gene sequence assembly is provided in [30].

In the experiments conducted, CAP3 gene sequence assembly is directly adopted in the Map phase of *PMR* and Hadoop. In the Reduce phase, result aggregation is considered. Performance evaluation of CAP3 execution on *PMR* and Hadoop frameworks* Homo sapiens* chromosome 15 is considered as a reference. Genome sequences of various sizes are considered as queries and submitted to Azure cloud platform in the experiments. Query sequences for experiments are considered in accordance to [30]. CAP3 experiments conducted with query genomic sequences (BAC datasets) are summarized in Table 2. All four experiments are conducted using CAP3 algorithm on the Hadoop and *PMR* frameworks. Observations are retrieved through a set of log files generated during Map and Reduce phase execution on Hadoop and *PMR*. Using the log files total makespan, Map worker makespan and Reduce worker makespan of Hadoop and *PMR* are noted for each experiment. It must be noted that the initialization time of the VM cluster is not considered in the computing makespan as it is uniform in *PMR* and Hadoop owing to similar cluster configurations.

**Figure 8:**
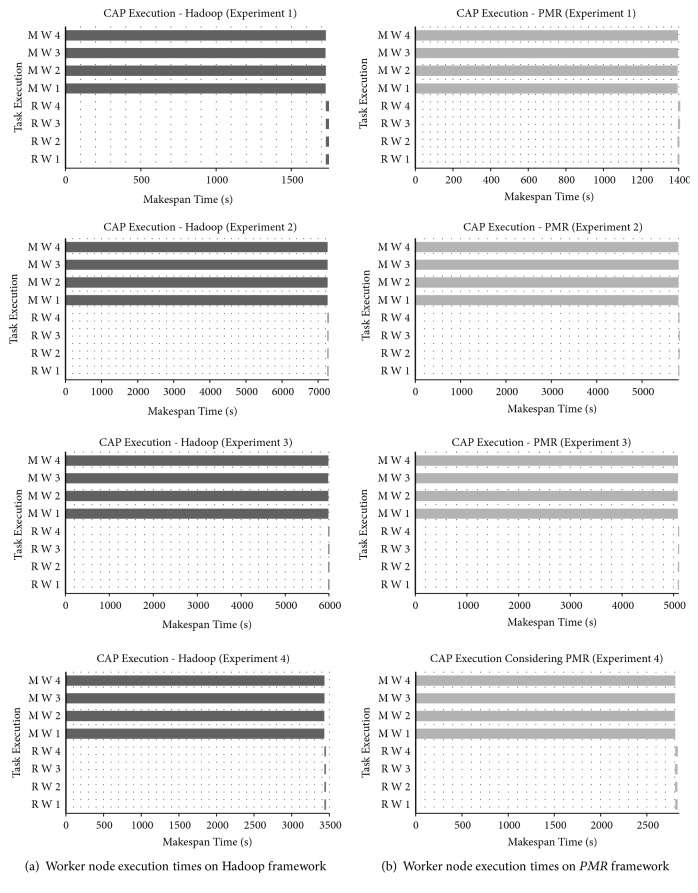
CAP3 sequence assembly execution makespan of the Map and Reduce worker nodes. (a) On Hadoop cluster of 4 nodes. (b) On *PMR* cluster of 4 nodes.

Task execution times of Map and Reduce worker nodes observed for CAP3 experiments conducted on Hadoop and *PMR* frameworks are shown in Figure 8.  Figure 8(a) represents results obtained for Hadoop and Figure 8(b) represents results obtained on *MR*. Execution times of Map worker nodes are far greater than execution times of Reduce worker nodes as CAP3 algorithm execution is carried out in the Map phase and result accumulation is considered in the Reduce phase. Parallel execution strategy (utilizing 4 computing cores available with each Map worker) of CAP3 algorithm on *PMR* enables lower execution times when compared to Hadoop Map worker nodes. Average reduction of execution time in Map workers of *PMR* reported is 19.33%, 20.07%, 15.09%, and 18.21% in CAP3 experiments conducted when compared to Hadoop Map worker average execution times. In *PMR*, the Reduce workers are initiated as soon as two or more Map worker nodes have completed their tasks which is visible from Figure 8(b). Sequential processing strategy (i.e., Map workers first and then Reduce workers execution) of worker nodes in the Hadoop framework is evident from Figure 8(a). Execution time of Reduce worker nodes in *PMR* is marginally higher by about 15.42% than those of Hadoop. Waiting for all Map worker nodes to complete their tasks is a primary reason for the marginal increase in Reduce worker execution times in *MR*.

**Figure 9:**
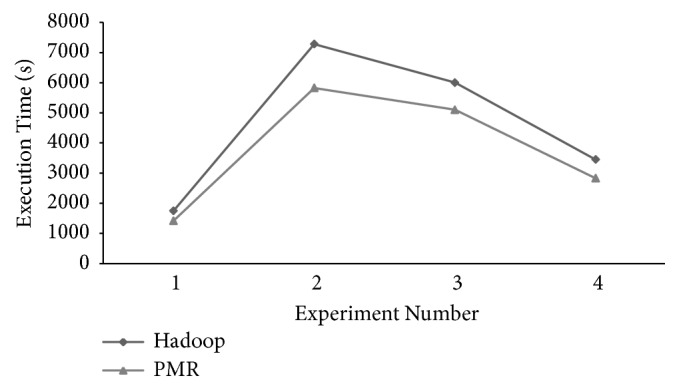
CAP3 sequence assembly total makespan time observed for experiments conducted on *PMR* and Hadoop frameworks.

The total makespan observed in CAP3 experiments executed on the Hadoop and *PMR* frameworks is presented in Figure 9. Superior performance in terms of Reduce makespan times of *PMR* is evident when compared to Hadoop. Though a marginal increase in Reduce worker execution time is reported, overall execution time, i.e., total makespan of *PMR*, is less when compared to Hadoop. A reduction of 18.97%, 20%, 15.03%, and 18.01% is reported for the four experiments executed on the *PMR* framework when compared to similar experiments executed on the Hadoop framework. Average reduction of the total makespan across all experiments is 18% proving superior performance of *PMR* when compared to the Hadoop framework. Makespan time for experiment 2 is greater than other experiments as the number of differences considered in CAP3 is 17 larger than values considered in other experiments. Similar nature of execution times is reported in [29] validating CAP3 execution experiments presented here.

**Figure 10:**
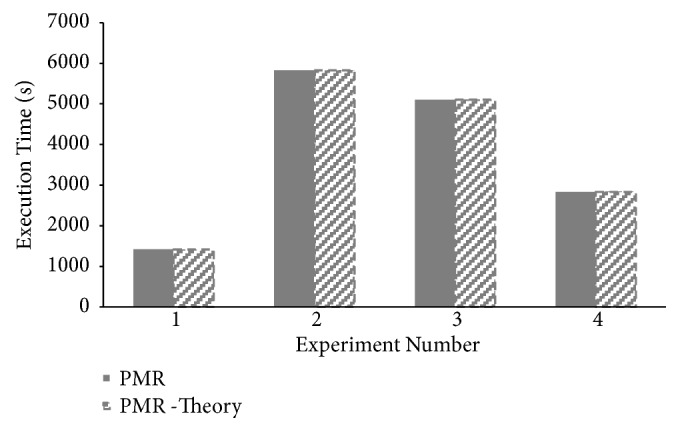
Correlation between theoretical and practical makespan times for CAP3 sequence assembly execution on PMR framework.

Theoretical makespan of *PMR* for all four CAP3 experiments is computed using (18). Comparison between theoretical and experimental makespan values is presented in Figure 10. Minor differences are reported between practical and theoretical makespan computations proving correctness of *PMR* makespan modeling presented.

The results presented in this section prove that CAP3 sequence assembly execution on the *PMR* cloud framework developed exhibits superior performance when compared to similar CAP3 experiments executed on the existing Hadoop cloud framework.

### 4.3. DeepBind Analysis to Identify Binding Sites

In recent times, deep learning techniques have been extensively used for various applications. Deep learning techniques are adopted predominantly when large amounts of data are to be processed or analyzed. To meet large computing needs of deep learning techniques, GPU are used. Motivated by this, the authors of the paper consider very recent state-of-the-art “DeepBind” biomedical application execution on a cloud platform. To the best of our knowledge, no such attempt to consider cloud platforms for DeepBind execution has been reported.

Alternative splicing, transcription, and gene regulations biomedical operations are dependent on DNA- and RNA-binding proteins. DNA- and RNA-binding proteins described using sequence specificities are critical in identifying diseases and deriving models of regulatory processes that occur in biological systems. Position weight matrices are used in characterizing specificities of a protein. Binding sites on genomic sequences are identified by scanning position weight matrices over the considered genomic sequences. DeepBind is used to predict sequence specificities. DeepBind adopts deep convolutional neural networks to achieve accurate prediction. Comprehensive details and sequence specificity prediction accuracy of the DeepBind application are available in [27].

**Figure 11:**
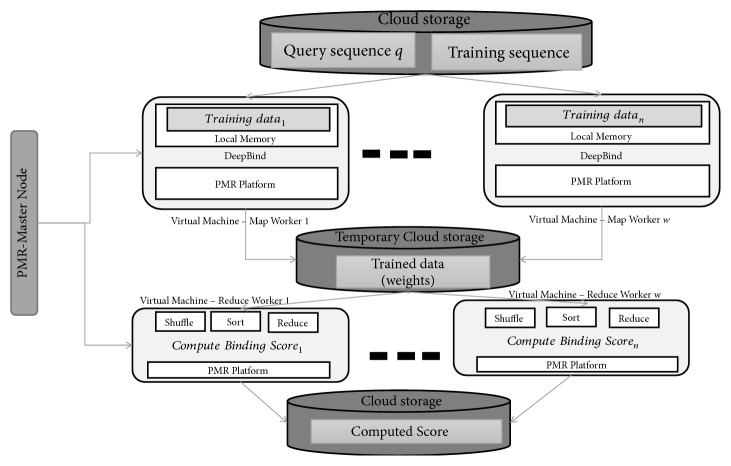
DeepBind PMR framework.

DeepBind is developed using a two-phase approach, a training phase and testing phase. Training phase execution is carried out using Map workers in the Hadoop and *PMR* frameworks. The trained weights are stored in the cloud memory for further processing. The testing phase of DeepBind is carried out at the Reduce stage in the Hadoop and *PMR* frameworks. Execution strategy of DeepBind algorithm on the *PMR* framework is shown in Figure 11. DeepBind application is developed using the code provided in [27]. For performance evaluation on Hadoop and *PMR* only testing phase is discussed (i.e., Reduce only mode). A custom cloud cluster of one master node and six worker nodes is deployed for DeepBind performance evaluation. A similar cloud cluster for the Hadoop framework is considered. The experiment conducted to evaluate the performance of DeepBind on the Hadoop and *PMR* frameworks considers a set of six disease-causing genomic variants obtained from [27]. The disease-causing genomic variants to be analyzed using DeepBind are summarized in Table 3. DeepBind analysis is executed on the Hadoop and *PMR* frameworks deployed on a custom cloud cluster. Log data generated is stored and used in further analysis.

**Figure 12:**
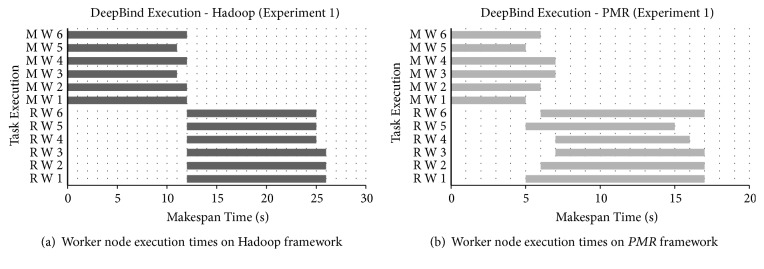
DeepBind execution makespan of the Map and Reduce worker nodes. (a) On Hadoop cluster of 6 nodes. (b) On *PMR* cluster of 6 nodes.

**Figure 13:**
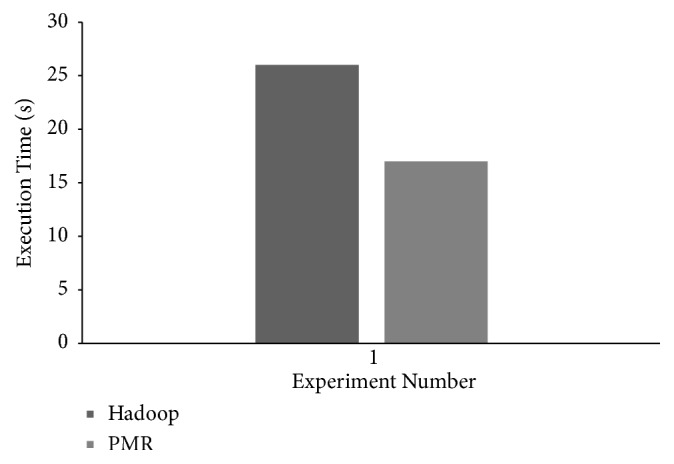
DeepBind analysis total makespan time observed for experiments conducted on *PMR* and Hadoop frameworks.

**Figure 14:**
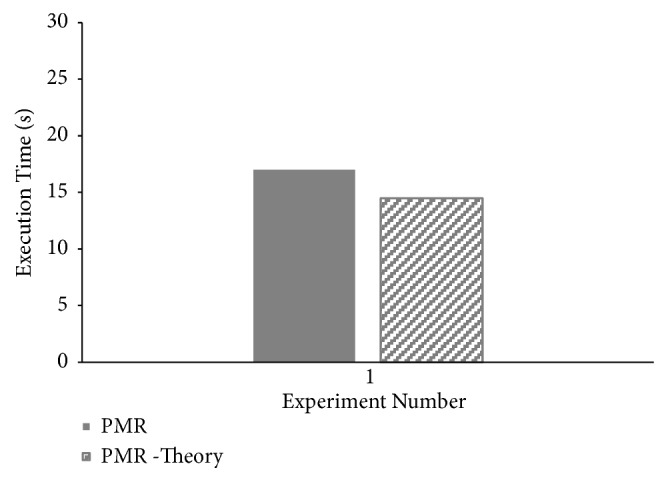
Correlation between theoretical and practical makespan times for DeepBind analysis execution on PMR framework.

The results obtained to demonstrate the performance of six worker cluster nodes of Hadoop and *PMR* during Map and Reduce phase execution are shown in Figure 12. Performance is presented in terms of task execution times observed per worker node. Considering Hadoop worker nodes execution times of each node during the Map and Reduce phase is shown in Figure 12(a). The execution time observed for each *PMR* worker node during the Map and Reduce phases is shown in Figure 12(b). In the Map phase execution, the genomic variants to be analyzed are obtained from the cloud storage and are accumulated based on their identities defined [27]. The query sequences of disease-causing genomic variants to be analyzed are split for parallelization. In the Reduce phase, the split query sequences are analyzed and results obtained are accumulated and stored in the cloud storage. Map workers in *PMR* exhibit better performance and an average execution time reduction of 48.57% is reported when compared to Hadoop Map worker nodes. Execution time of the six Reduce worker nodes in Hadoop and *PMR* is greater than Map workers as DeepBind analysis and identification of potential binding sites is carried out during this phase. Parallel execution strategy of Reduce worker nodes is clear from Figure 12(b). The Reduce phase in *PMR* commences after 5 seconds once Map worker node 1 (MW1) and Map worker node 5 (MP5) have completed their task. In Hadoop that adopts a sequential approach, the Reduce phase is initiated after all worker nodes have completed their tasks. Parallel execution of DeepBind analysis utilizing all 4 computing cores available with Reduce worker nodes and parallel initiation of the Reduce phase in *PMR* enable average Reduce execution time of 22.22% when compared to Hadoop Reduce worker nodes. The total makespan observed for DeepBind experiment execution on the Hadoop and *PMR* cloud computing platforms is shown in Figure 13. Total makespan reduction of 34.62% is achieved using the *PMR* framework when compared to the Hadoop framework. Analysis results similar to [27] are reported for DeepBind analysis on the Hadoop and *PMR* frameworks. The theoretical makespan computed using (18) for *PMR* is compared with the practical value observed in the experiment and the results obtained are shown in Figure 14. A minor variation between theoretical and practical values is observed. The variation observed is predominantly due to application dependent multiple cloud memory access operations. Based on results obtained for DeepBind analysis, it is evident that performance on the *PMR* framework is far superior to its execution on the existing Hadoop framework.

On the basis of biomedical applications considered for performance evaluation and results obtained, it is evident that the proposed *PMR* framework exhibits superior performance when compared to its existing Hadoop counterpart. In BLAST, the Map and Reduce phases are utilized. In CAP3 application, the Map phase plays a predominant role. In DeepBind application analysis is carried out in the Reduce phase. The proposed *PMR* cloud computing framework is robust and is capable of the dynamic biomedical application scenarios presented: deployment of *PMR* on public and custom cloud platforms. In addition, *PMR* exhibits low execution times and enables effective cloud resource utilization. Low execution times enable cost reduction, always a desired feature.

## 5. Conclusion and Future Work

The significance of cloud computing platforms is discussed. The commonly adopted Hadoop MapReduce framework working with its drawbacks is presented. To lower execution times and enable effective utilization of cloud resources, this paper proposes a *PMR* cloud computing platform. A parallel execution strategy of the Map and Reduce phases is considered in the *PMR* framework. The Map and Reduce functions of *PMR* are designed to utilize multicore environments available with worker nodes. The paper presents the proposed *PMR* framework architecture along with makespan modeling. Performance of the *PMR* cloud computing framework is compared with the Hadoop framework. For performance evaluation, computationally heavy biomedical applications like BLAST, CAP3, and DeepBind are considered. Average overall makespan times reduction of 38.92%, 18.00%, and 34.62% is achieved using the *PMR* framework when compared to the Hadoop framework for BLAST, CAP3, and DeepBind applications. The experiments presented prove the robustness of the *PMR* platform, its capability to handle diverse applications, and ease of deployment on public and private cloud platforms. The results presented through the experiments conducted prove the superior performance of *PMR* against the Hadoop framework. Good matching is reported between the theoretical makespan of the *PMR* presented and experimental values observed. In addition, adopting the *PMR* cloud computing framework also enables cost reduction and efficient utilization of cloud resources.

Performance study considering cloud cluster with many nodes, additional applications, and security provisioning to cloud computing framework is considered as the future work of this paper.

## Figures and Tables

**Table 1:** Information of the Genome Sequences used as queries considering equal section lengths from *Homo sapiens* chromosome 15 as a reference.

**Experiment Id**	**Query genome**	**Query genome size** **(bp)**	**Database sequence**	**Reference genome size** **(bp)**
1	NT_007914	14866257	*Drosophila* database	122,653,977
2	AC_000156	19317006	*Drosophila* database	122,653,977
3	NT_011512	33734175	*Drosophila* database	122,653,977
4	NT_033899	47073726	*Drosophila* database	122,653,977
5	NT_008413	43212167	*Drosophila* database	122,653,977
6	NT_022517	90712458	*Drosophila* database	122,653,977

**Table 2:** Information of the Genome Sequences used in CAP3 experiments.

**Experiment Number**	**Dataset**	**GenBank accession number**	**Number of reads (bp)**	**Average Length of reads (bp)**	**Length of provided sequences (bp)**
1	203	AC004669	1812	598	89779
2	216	AC004638	2353	614	124645
3	322F16	AF111103	4297	1011	159179
4	526N18	AF123462	3221	965	180182

**Table 3:** Information of the disease-causing genomic variants used in the experiment.

**Experiment 1**		
**Case study**	**Genome variant**	**Experiment details**
1	SP1	A disrupted SP1 binding site in the LDL-R promoter that leads to familial hypercholesterolemia
2	TCF7L2	A cancer risk variant in a MYC enhancer weakens a TCF7L2 binding site
3	GATA1	A gained GATA1 binding site that disrupts the original globin cluster promoters
4	GATA4	A lost GATA4 binding site in the BCL-2 promoter, potentially playing a role in ovarian granulosa cell tumors
5	RFX3	Loss of two potential RFX3 binding sites leads to abnormal cortical development
6	GABPA	Gained GABP-*α* binding sites in the TERT promoter, which are linked to several types of aggressive cancer

## Data Availability

The data is available at the National Center for Biotechnology Information. (2015). [Online]. Available: http://www.ncbi.nlm.nih.gov/
